# Development of Motivate4Change Using the Intervention Mapping Protocol: An Interactive Technology Physical Activity and Medication Adherence Promotion Program for Hospitalized Heart Failure Patients

**DOI:** 10.2196/resprot.4282

**Published:** 2015-07-20

**Authors:** Rony Oosterom-Calo, Saskia J te Velde, Wim Stut, Johannes Brug

**Affiliations:** ^1^Philips ResearchBriarcliff Manor, NYUnited States; ^2^EMGO Institute for Health and Care Research and the Department of Epidemiology and Biostatistics, VU University Medical CenterAmsterdamNetherlands; ^3^Philips ResearchEindhovenNetherlands; ^4^EMGO institute for Health and Care Research and the Department of Epidemiology and Biostatistics, VU University Medical CenterAmsterdamNetherlands

**Keywords:** heart failure, self-care, self-management, interactive technology, medication adherence, physical activity, computer tailoring, intervention mapping, hospital

## Abstract

**Background:**

It is important that heart failure (HF) patients adhere to their medication regimen and engage in physical activity. Evidence shows that adherence to these HF self-management behaviors can be improved with appropriate interventions.

**Objective:**

To further promote medication adherence and physical activity among HF patients, we developed an intervention for hospitalized HF patients.

**Methods:**

The intervention mapping protocol was applied in the development of the intervention. This entailed performing a needs assessment, defining change objectives, selecting determinants and strategies, and developing the materials.

**Results:**

The resulting intervention, Motivate4Change, makes use of interactive technology and provides HF patients with personalized feedback and advice. Specific change objectives were defined. The relevant behavioral determinants for the physical activity program were practical knowledge on physical activity performance and self-efficacy for, and perceived benefits of, physical activity. For medication-taking, the selected determinants were practical knowledge on medication-taking, perceived barriers to medication-taking, beliefs about the necessity and harm regarding the medication prescribed, and beliefs about overprescribing and harm of medication in general. The change objectives and behavior change determinants were translated in feedback and advice strategies in an interactive technology program that included tailored feedback and advice, and role models in videos in which the behaviors and overcoming barriers were demonstrated. Relevant stakeholders were involved in the interventions development process. The intervention was pretested among HF patients and adjustments were made accordingly.

**Conclusions:**

The interactive technology physical activity and medication adherence promotion program for hospitalized HF patients was systematically developed using the intervention mapping protocol and was based on the available theory and evidence regarding HF self-management behavior change. The intervention’s efficacy is yet to be determined in evaluation research.

## Introduction

### Background

Heart failure (HF) is a chronic condition, affecting primarily older patients [1]. Although HF is usually irreversible, it can be effectively managed with medication and behavioral treatment, including physical activity [2]. HF medications have proven effective in various clinical trials [2], and medication adherence among HF patients is related to event-free survival [3]. However, in studies that use objective measurement techniques, low medication adherence rates are demonstrated [4]. The physical activity recommendation for HF patients specifies performance of at least 30 minutes of moderate intensity physical activity daily [5] . Performance of physical activity among HF patients was found to be related to reduced readmission rates in contrast to the other studied nonpharmacological self-care behaviors (daily weighing, fluid restriction, low-salt diet) [6]. Despite the potential benefits, HF patients report insufficient rates of physical activity [7,8]. Interventions that promote medication-taking and physical activity among HF patients are therefore warranted.

Interactive technology is a promising channel for delivery of patient education and health promotion interventions since it may increase interest and recall of information. Using auditory, visual, and interactive learning strategies, knowledge can be transferred to patients with various learning capabilities [9]. Due to constraints of the medical system, currently not all patients may be provided with education, and interactive technology solutions are a feasible alternative [9]. HF nurses name various barriers to provision of patient education in the hospital, including insufficient teaching materials [10]. So far, face-to-face or telephone-based education and counseling programs for HF patients demonstrate promising results [11-13], but interactive, technology-based education and counseling interventions for HF inpatients are scarce. A CD-ROM-based educational program has been developed for HF patients for the hospital setting [14] focusing on providing generic information to patients by displaying animations, photos, and voice-overs. This program has demonstrated similar but not superior results as standard education [15] and patient acceptance of the technology [16]. It could be that addressing patient motivation to engage in the behaviors and tailoring the information to individual needs and barriers may be beneficial in comparison with standard education, and interactive technology provides the opportunity to do that.

When designing an intervention, the setting and timing for intervention delivery should be carefully considered. Hospitalization has been referred to as a teachable moment [17] because patients may be more motivated to change their behavior soon after being confronted with their disease. Moreover, many patients can be reached during hospitalization with relatively little effort (as opposed to, for instance, a home visit by a community nurse). An HF nurse typically provides education in the hospital to patients before their discharge from the hospital [18]. Previous work suggests that hospital nurses may not always provide effective education, because of lack of time and/or training, that individualizing content to patients may be one particular area in which nurses could use support, and that nurses do not have sufficient materials (teaching aids) to support them [10]. This suggests that although there is potential to reach many patients in the hospital, the education being provided by hospital nurses may not be optimal.

Interactive technology health behavior promotion interventions hold the potential for individualizing content, as the expertise of the health educator is documented in the interactive program and can be used by nurses to supplement their education. With an interactive technology program, patients can use the entire hospitalization period to become informed regarding their self-care because the nurse does not necessarily have to be present, thereby increasing the learning potential. In the current situation, patients are educated by HF nurses about self-care typically just before they are discharged from the hospital and need to start performing the behaviors when they at home. The transition from the hospital to the home environment is reported to be a point of confusion and miscommunication [19], and interventions that assist in supporting learning and reducing the potential confusion are desired.

### Objectives

**Figure 1:**
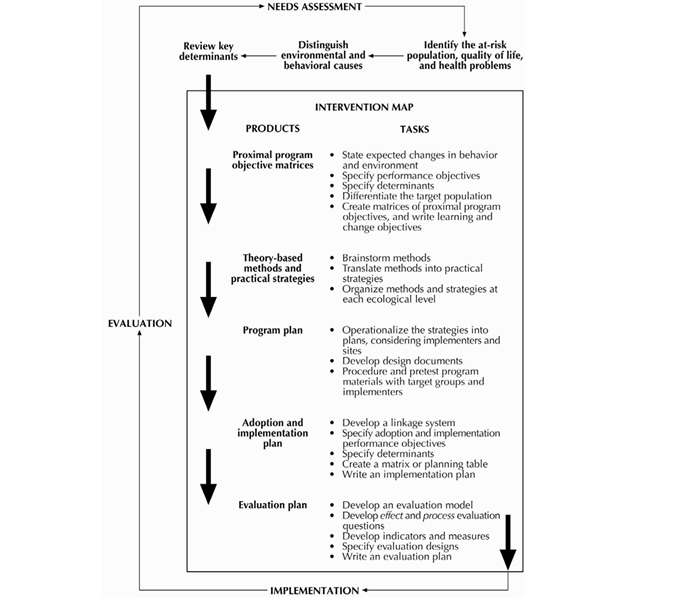
The intervention mapping protocol. Source: Bartholomew et al (2001) [21].

We aimed to develop an individually tailored interactive technology program for the promotion of medication adherence and physical activity among HF patients, for delivery in the hospital setting. The goal of the program, which we named Motivate4Change, is to educate and motivate patients to engage in physical activity and medication adherence after their discharge from the hospital. To increase the level of confidence that the intervention we develop would be efficacious, we worked according to the intervention mapping protocol. The intervention mapping protocol is a stepwise method (Figure 1) used to develop interventions systematically using relevant theory and evidence [20]. By going through the steps and creating matrices, decisions are made regarding the specific behavioral change objectives and how to achieve these changes by identifying theory and evidence-based strategies and behavior change techniques. This allows targeting the behavioral determinants and using behavior change strategies that are most likely to affect the desired outcomes. In the current paper we report on the design of Motivate4Change using an intervention mapping approach.

## Methods

The intervention mapping protocol specifies six steps for the development of theory and evidence-based health promotion interventions [21]. We implemented the first four steps in the development of Motivate4Change, including (1) the needs assessment, (2) the definition of the performance objectives, selection of behavioral determinants, and definition of matrices of change objectives, (3) selection of methods and behavior change strategies, and (4) the translation of these into an actual intervention program. To define the program objectives, relevant literature and medical guidelines were consulted. This led to a definition of the performance objectives. To define the important and changeable determinants for these performance objectives, systematic literature reviews were conducted [22,23]. Based on the performance objectives and selected behavioral determinants, matrices of change objectives were created. Strategies for behavior change were selected by consulting literature and behavioral theories to identify strategies that target behavioral constructs. After these steps were taken, the intervention program and materials were devised. These included the program structure and sequence, assessments, tailored messages, and functional research prototype of the program.

## Results

### Step 1: Needs Assessment

#### Management of Heart Failure

Effective management of HF is needed to curb adverse outcomes. Reported HF prevalence rates in men are 8 per 1000 at age 50 to 59 years, increasing to 66 per 1000 at ages 80 to 89 years (similar values (8 and 79 per 1000) were noted in women) [24]. Poor health outcomes include high mortality [25], frequent acute episodes leading to hospital readmissions [26], and low levels of quality of life [27] and wellbeing [28] for patients and caregivers. In addition, the costs of HF are high, approximately 1%-2% of the total health care budget in developed countries, primarily due to the readmissions [29]. The management of HF primarily includes medication prescriptions and self-care behavior although in some cases surgery may be possible [2].

#### Promotion of Medication Adherence

Evidence-based pharmacological treatment is common practice in the management of HF. Commonly prescribed drugs include diuretics, angiotensin-converting enzyme inhibitors, and beta blockers, among others [30]. Medication adherence, when measured with the Medication Event Monitoring System (MEMS), was found to be related to event-free survival [3]. However, for the medications to be effective, patients need to take them as prescribed. Although research on medication adherence using self-report measures demonstrates that adherence to HF medication is adequate [8], studies that use more objective measurement techniques such as claims data demonstrating prescription fillings [31,32] and MEMS data [4] demonstrate low adherence rates and thus room for improvement.

#### Promotion of Physical Activity

It is recommended that HF patients perform moderate intensity physical activity for at least thirty minutes daily [5]. There is evidence that performance of physical activity among HF patients can lead to a reduction in readmission and mortality rates [33]. Despite the promising effects, numerous studies show that adherence to physical activity recommendations is inadequate among HF patients [2,7,34,35]. In addition, health care providers do not always promote physical activity among HF patients. Reasons include lack of knowledge on behalf of the health care providers and financial reasons [36]. Education on self-care behaviors, including physical activity, is often not provided for hospitalized patients due to lack of time [18].

#### Needs in the Hospital Setting

As previously mentioned, Motivate4Change was developed for delivery in the hospital setting, although patients would perform the self-care behaviors after they are back at home. As such, we assessed the needs of patients during hospitalization, when they would be engaging in the intervention. Patients’ needs for completion of an interactive technology health behavior promotion program may be different in the hospital setting than in the home setting. The needs assessment for the hospital setting revealed that the short length of stay and high symptom acuity level [10] are challenges that are specific to the hospital setting. These challenges could lead to patients experiencing negative emotions and feeling less focused and more confused, in addition to having only a short period of time to engage in the intervention. Based on these identified issues, specific requirements for the intervention were defined including a short length for the intervention program and clear and simple content and user interface. An intervention meeting these requirements was considered more likely to be suitable in light of the state of confusion and symptom acuity that patients experience in the hospital.

Based on the needs assessments, we defined the aim of the intervention. For a health behavior promotion intervention provided in the hospital setting, the behavioral objectives can only be achieved some time after the provision of the intervention; it is unlikely that patients will take their medications independently or perform physical activity during hospitalization. Therefore, the aim of Motivate4Change was that “patients have the *intention* to take their medications as prescribed and increase the amount of physical activity they perform.” According to the theory of planned behavior (TPB) [37], intentions to perform a behavior are the closest factor to actual behavioral performance. We found in the scientific literature that many patients perform very little physical activity, and this information was also verified by a cardiologist and a HF nurse. Based on their input, we defined the physical activity aim loosely and not specifically according to the recommendation for this patient population because for patients who perform very little physical activity, an increase to the recommended level of at least 30 minutes of moderate intensity activity daily was perceived an unrealistic aim for a brief intervention, to be delivered only during hospitalization.

### Step 2: Definition of Performance Objectives, Selection of Behavioral Determinants and Creation of Matrices of Change Objectives

#### Definition of Performance Objectives

Performance objectives are the specific cognitions and/or actions required towards intentions for behavior change. Complex behaviors often need to be changed by addressing their components and making a change in each of these components. The performance objectives (Textbox 1), corresponding to the program objectives mentioned above, were cognitive because the program objective was cognitive.

**Performance objectives for intentions to take medications as prescribed and to perform more physical activity.**

Performance objectives for intentions to take medications as prescribed:

- Patients describe how to take their medications
- Patients identify solutions to their barriers to taking medications
- Patients specify counterarguments to their concerns to taking medications
- Patients specify reasons why their medications are necessary

Performance objectives for intentions to perform more physical activity:

- Patients describe how to perform physical activity
- Patients identify solutions to their barriers to performing physical activity

#### Selection of Behavioral Determinants

Health promotion interventions should address the most important and modifiable behavioral determinants so that the behaviors are influenced and the health problem diminished [20]. The identification of behavioral determinants and the translation of these determinants into intervention strategies and techniques should be guided and informed by behavioral theories as well as scientific evidence. Since the targeted delivery setting was the hospital, determinants had to be changeable in a brief intervention in the hospital setting. Theoretical health behavior models and scientific literature were consulted in the selection of determinants.

##### Theoretical Indications

After reviewing prominent health behavior theories, two were selected to be used for guidance in the selection of determinants: TPB [38] and social cognitive theory (SCT) [39]. These were selected in part because they describe cognitive determinants of behavior that were deemed changeable in a brief intervention and are widely used in the design of new health promotion interventions [40].

The concept of perceived self-efficacy, or one’s confidence in being able to perform a specific behavior, is central in SCT [41]. Another key concept is outcome expectations. SCT postulates that behavioral motivation is generated through cognitive representations of future outcomes of performing the behavior. According to TPB, three cognitive factors influence intentions for behavior: behavioral attitude (ie, one’s beliefs regarding the behavior), subjective norms which results from someone’s normative beliefs, and perceived behavioral control, which is based on control beliefs and considered similar to the self-efficacy concept of SCT.

##### Scientific Literature

We conducted two reviews in which the determinants of self-care [22] and adherence to medication [23] were investigated. Self-care consists of multiple behaviors including self-care management; self-care maintenance; sodium, fluid, and alcohol intake restriction; physical activity; smoking cessation; monitoring signs and symptoms; and keeping follow-up appointments. Overall, important determinants were perceived benefits and barriers (specifically related to sodium intake restriction) and type-D personality (specifically related to keeping follow-up appointments). However, we found inconsistent evidence of any of the determinants investigated in the included studies in relation to increase in physical activity. Having been hospitalized in the past was found in the review to be related to higher adherence to medication. Since we found no evidence of determinants related to increasing physical activity for HF patients and past hospitalization was not a determinant that we could easily address in our short intervention, we had to expand our search and look for determinants of increasing physical activity and medication adherence in general.

Previous work [42,43] outlines a wide range of potential barriers to medication adherence, including aging-related factors such as cognitive and physical declines, social and economic factors [43], and patients’ health condition and treatment [42]. Most of these determinants were deemed not suitable for a brief intervention in a hospital setting because more time and effort would be required to observe a change in them. Beliefs about medications, which are more suitable to target within a brief intervention, are also important determinants of medication adherence [44]. Beliefs may be about side effects of medications, efficacy of medications as well as negative views about medication in general. Knowledge and understanding may also be important for medication adherence because HF patients are prescribed an average of eight different medications and they usually need to take medication more than once per day [45].

Self-efficacy is an important determinant of physical activity in the general population [46] and among older adults [47,48]. There is also evidence that targeting self-efficacy in interventions is an effective means to increase engagement in physical activity [49]. Specific barriers for physical activity that HF patients may feel a lack of confidence overcoming include physical symptoms [50], environmental influences (eg, weather, resources), expectations of others (eg, encouragement of relatives, interest and advice of others), mental outlook (eg, no self-identification as active, lack of motivation for physical activity) and fluctuating health (eg, HF symptoms) [51].

In sum, three determinants of medication adherence were selected, which were deemed important and suitable for targeting in a brief intervention: practical knowledge, self-efficacy for taking medication, and beliefs about medication. Two determinants for physical activity were selected: practical knowledge and self-efficacy to perform the activity.

#### Creation of Matrices of Change Objectives

Next, performance objectives for the program objectives were created. These are a set of subcognitions, and a change in those would lead to a change in the program objective (Table 2). Finally, the selected determinants and the performance objectives were integrated in matrices of change objectives (Table 2), which demonstrate what is necessary in terms of behavior or cognition to achieve the performance objectives. The change objectives were used to develop the content of the intervention materials.

**Table 2:** A selection of matrices of change objectives for the five performance objectives.

Performance objective	Knowledge	Self-efficacy	Beliefs
Patients are willing to take their medications	Patients describe the prescriptions of the doctor	Patients express their confidence they are able to follow the doctor’s prescriptions	Patients believe that taking the prescribed medications correctly can make them feel better
	Patients describe how they should take their medication	Patients express their confidence that they can take their medications every day	Patients believe that taking medication every day is necessary for them
Patients identify solutions to their barriers to taking medications	Patients recognize the side effects of medications	Patients express their confidence about what to do if side effects occur	Patients believe that if they have side effects this does not mean they should stop taking medications without a consultation
	Patients know which types of memory aids are available	Patients know where they can acquire memory aids	Patients believe that memory aids can be effective in helping them take their medications
Patients are willing to perform physical activity	Patients know they should perform physical activity on most days of the week	Patients identify activities that are enjoyable to them, which they are confident they can perform	
Patients describe how to perform physical activity	Patients know they should perform moderate intensity physical activity	Patients describe the ‘talk test’ for measuring intensity of physical activity	
Patients identify solutions to their barriers to performing physical activity	Patients list appropriate activities when the weather is bad	Patients express confidence that when experiencing breathlessness or fatigue, they can rest and perform activity again when feeling better	

### Step 3: Selection of Strategies for Behavior Change

#### Tailored Health Communication

Health information which is tailored to patients’ relevant psychosocial characteristics has been found to be more motivating than generic health information [52]. Tailoring can be achieved with a computer system and provided through interactive technology and is typically done by assessing patients on relevant characteristics and providing information according to the assessment results [53]. Some of the reported mechanisms for the superior efficacy of tailored information over generic information include personalization of the information, better exposure to the information, more intensive cognitive processing, greater relevance of the information provided to the individual receiving it, and self-evaluation properties of the feedback received [54]. The information in Motivate4Change was tailored to individual patients based on the behavioral determinants identified in the previous step. Specifically, the information was tailored to individual knowledge levels and existence of barriers which may reduce self-efficacy; in the medication adherence module, the information was also tailored to problematic beliefs about medications. Therefore, patients received personalized written and video messages [55].

#### Instruction

Instruction on how to perform the behavior, including provision of information about the behavior and expected outcome was another strategy used to improve self-efficacy and knowledge. In addition, instruction was provided regarding solutions to overcome barriers. It has been shown that both active and inactive older adults have barriers for performing physical activity, but active older adults recognize solutions to overcome their barriers while inactive older adults do not recognize solutions [56].

#### Vicarious Experience

Self-efficacy, as outlined by SCT, can be promoted through three channels: performance accomplishments, vicarious experience, and verbal persuasion [41]. For hospitalized HF patients the first does not apply, because they do not perform the behaviors while in the hospital. Vicarious experience includes seeing others perform the behaviors, leading people to persuade themselves that if others can do it, they should be able to at least partially achieve it too [41]. This strategy was incorporated in the current intervention with a video displaying an actor playing the role of a typical HF patient successfully performing physical activity and taking medication.

#### Persuasion Techniques

To persuade patients to perform the behavior, we incorporated references to authority [57] whereby people are persuaded to perform a behavior because an authority figure recommends performing this behavior. In Motivate4Change, a professor in cardiology appeared in the videos and emphasized the importance of physical activity and medication adherence for HF patients.

#### Empathy

In order to increase patient acceptance of the messages being communicated, empathy was an additional strategy used in the current intervention. Hospitalization may be a difficult time for patients [58], and therefore empathic communication was deemed suitable. Also, some of barriers that patients were asked about may be sensitive and/or difficult for patients, and it was therefore appropriate to address them with an empathic tone. Empathy is one of the main principles in motivational interviewing [59]. Empathic sentences were incorporated in the messages, such as “Many patients feel that…” or “It is understandable that…”

### Step 4: Producing Program Components and Materials

**Figure 2:**
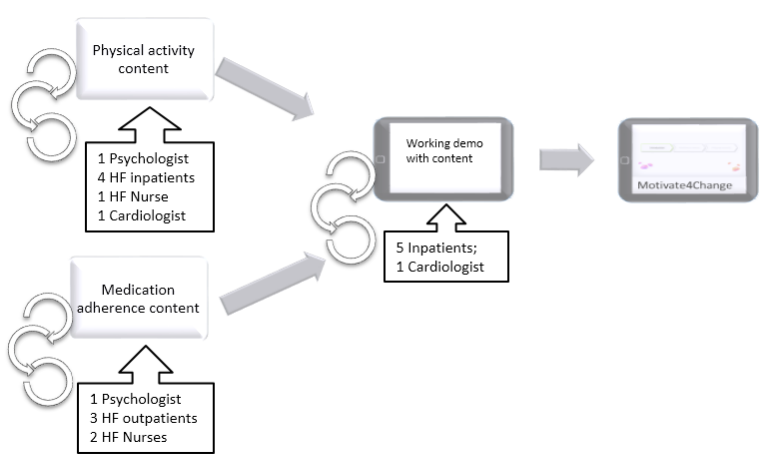
The iterative process of the intervention content development.

In this step the program plan was developed, including the scope and sequence, and the program materials were formulated. Relevant stakeholders were involved in the development of the intervention materials, including cardiologists, HF nurses, and patients (Figure 2). The user interface was designed taking into account the presumed needs of the target group in terms of usability (eg, having a clear indication how to go forwards and backwards in the program, having a large font size), and usability sessions were conducted, leading to an identification of issues which were addressed in the next version. In preparation for the usability session, observation sheets were prepared in which various relevant usability attributes such as learnability, efficiency, and simplicity were specified; for each attribute specific indicators were specified, which could be observed and recorded by the researcher. The researcher observed patients while they were using the research prototype and took extensive notes while completing the usability sheets.

The messages which were created for Motivate4Change addressed the listed determinants and corresponding change objectives. Messages were reviewed by nurses to check for medical correctness and suitability for patients. Feedback sessions with patients took place, in which patients were asked to read the message texts out loud so the researchers could observe and note difficulties. Patients were asked to share their opinions on the content, and the feedback was incorporated (Figure 2). Specifically, based on patient input, messages related to specific barriers to perform the health behaviors were removed or added where appropriate. For example, although at first a message relating only to side effects as a barrier for patients was formulated, it was found in the feedback sessions that medications’ desired effects can also be a barrier for patients, as is the case with frequent urination when taking diuretics in order to reduce fluid buildup. Based on the literature, finding time to perform physical activity was identified as a barrier, but HF patients who participated in the feedback sessions remarked this is not a barrier for them, and the relevant message was thus removed. A cardiologist was asked to provide feedback on the final program and make sure the content was correct from a medical perspective.

**Figure 3:**
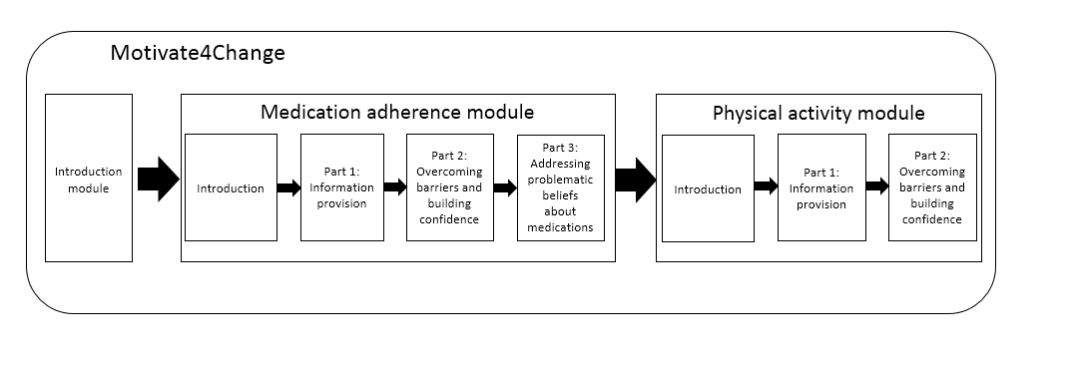
The Motivate4Change intervention structure.

**Figure 4:**
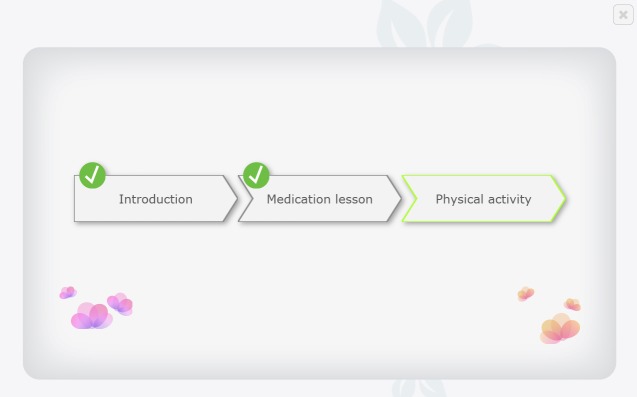
The Motivate4Change program menu.

**Figure 5:**
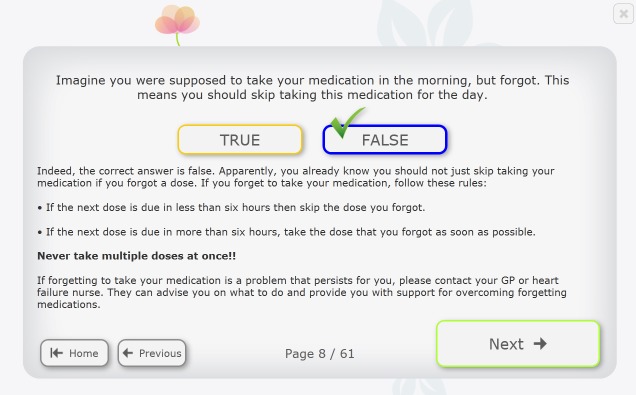
A medication knowledge item from Motivate4Change.

The resulting program included an introduction, which included an explanation of the intention of the program and a summary of the key take-away messages in relation to medication adherence and physical activity for HF patients, and two modules, the first on medication adherence and the second on physical activity (Figure 3). Medication adherence was deemed more urgent to address based on input from clinicians, and it was therefore placed first. The medication adherence module included three parts; one which was meant to increase practical knowledge on taking HF medications, another to assess barriers to taking HF medications and provide solutions to identified barriers, and a third part to assess problematic beliefs relating to medications and providing messages to address those problematic beliefs. The physical activity module had two parts; one aiming to increase practical knowledge on performing physical activity and the other another assessing barriers to performance and providing solutions to identified barriers. Within modules, patients are presented with videos followed by assessments and immediate tailored feedback messages (Figures 4 and 5), depending on their answers. The videos were from the Philips Motiva telehealth system.

Assessments for tailoring the content were based on the Beliefs about Medication Questionnaire [60] and the Self-Efficacy to Regulate Exercise Questionnaire [61]. The scales in the original questionnaires were changed to dichotomous scales, and two tailored messaged were constructed for each question. We added and removed items based on input from patients and professionals.

## Discussion

### Principal Findings

In the development of an intervention promoting medication adherence and physical activity targeting HF patients in the hospital setting, a systematic approach guided by intervention mapping was found useful and informative. This process resulted in an intervention, Motivate4Change, which targets knowledge, self-efficacy, and beliefs using a variety of strategies including tailored health communication. We believe Motivate4Change is likely to be effective because its content was guided by scientific literature and behavioral theories as well as input from the potential users. Specifically, this process has resulted in a working research prototype that would be ready for implementation at a hospital. A usability testing of the user interface was conducted demonstrating the intervention can be used by older HF patients. However, it would be necessary to also conduct formative user research investigating the needs and requirements surrounding the intervention’s implementation to ensure it is implemented appropriately.

Since intervention development an be time and resource intensive processes, it is imperative to develop interventions that are likely to be effective. To do this, researchers must compile the existing evidence and theory in order to make decisions relating to the intervention’s content and structure. As such, researchers may be overwhelmed by the abundance of scientific literature available. A systematic process such as intervention mapping could help them make more informed decisions in a structured and relatively easy-to-follow manner.

### Limitations

Although intervention mapping was a very useful tool in the development of Motivate4Change and increased our confidence in the effectiveness of the intervention, it had one drawback. Specifically, although intervention mapping was found useful and informative, it was also a time-consuming and lengthy process in the development of Motivate4Change. There were two limitations to the way in which we implemented the intervention mapping process. First, after discovering that there was not enough available information on the determinants of medication adherence and physical activity among HF patients, we did not conduct additional empirical evidence on this topic, for pragmatic reasons, and instead relied on available evidence from other, similar, populations. In addition, when selecting a digital intervention to address the need for promotion of self-care in the needs assessment stage, we did not assess if the relevant stakeholders have a need specifically for a digital intervention. Instead, we hypothesized such an intervention would be adequate and desirable due to its potential to address some of the observed needs of the stakeholders.

The resulting intervention, Motivate4Change, has a few limitations. Although we believe it would be effective in changing patient behavioral intentions if tested in a trial, it may be important to combine it with long-term behavior change and adherence support also at home or in an outpatient setting to increase its effectiveness. In a brief intervention for hospitalized patients, only a few change objectives and determinants could be addressed; therefore, we selected the three determinants that were most relevant and changeable according to our research. In addition, the performance objectives were largely restricted to passive performance or actions. The target environment for the intervention delivery is a hospital setting, and the intervention is mainly to help patients plan, prepare, and anticipate actions when back in their home environment. Therefore, the performance objectives were mostly indirect, focusing on describing and identifying specific actions.

One important aspect in the development of the Motivate4Change intervention was the early assessment of the needs of the intended users in the intended setting and the involvement of relevant stakeholders in the intervention development. In the needs assessment phase, it was found that patients the hospital setting are likely to have needs that are specific for this setting, which had consequences for the intervention content, sequence, and structure. Designing the intervention with an early consideration of the needs of the intended users in the intended setting resulted in an intervention that is likely to fit the intended setting, making it more likely that it will affect clinical outcomes if tested in a trial.

Motivate4Change was restricted in its focus because of the predefined setting it was to be implemented in, which reduced the degrees of freedom in applying intervention mapping to come to the best possible intervention. This means that the resulting intervention is likely suitable for the hospital setting, but possibly less effective than an intervention that would have been developed with the intention of having a positive effect on the behavioral outcome, regardless of the setting for intended implementation. Finally, although we believe that Motivate4Change is likely to be effective in changing HF patients’ physical activity and medication adherence, the effectiveness is yet to be demonstrated. To assess the effectiveness of an intervention, a randomized controlled trial would be necessary as a next step. Although at this moment a trial assessing the effectiveness of Motivate4Change is not planned, such a trial would consist of an intervention group receiving Motivate4Change during hospitalization and a control group receiving standard care during hospitalization.

### Conclusions

In sum, the current work describes the development of the Motivate4Change intervention aimed at promoting medication adherence and physical activity among HF patients. This intervention was developed by following intervention mapping protocol. It was developed for use in the hospital setting and as such would be suitable for implementation in the hospital setting after a formative user needs and environmental requirements study.

## Abbreviations

HF: heart failure
MEMS: medication event monitoring system
SCT: social cognitive theory
TPB: theory of planned behavior
